# Serum exosomal-annexin A2 is associated with African-American triple-negative breast cancer and promotes angiogenesis

**DOI:** 10.1186/s13058-020-1251-8

**Published:** 2020-01-28

**Authors:** Pankaj Chaudhary, Lee D. Gibbs, Sayantan Maji, Cheryl M. Lewis, Sumihiro Suzuki, Jamboor K. Vishwanatha

**Affiliations:** 1 Department of Microbiology, Immunology and Genetics, Graduate School of Biomedical Sciences, University of North Texas Health Science Center, 3500 Camp Bowie Blvd., Fort Worth, TX 76107 USA; 2 Simmons Comprehensive Cancer Center, University of Texas Southwestern Medical Center, Dallas, TX 75390 USA; 3 Department of Biostatistics and Epidemiology, School of Public Health, University of North Texas Health Science Center, Fort Worth, TX 76107 USA; 4 Texas Center for Health Disparities, University of North Texas Health Science Center, Fort Worth, TX 76107 USA

**Keywords:** Annexin A2, Exosomes, Breast cancer, Angiogenesis, And racial disparity

## Abstract

**Background:**

Limited information is available on biomarker(s) for triple-negative breast cancer (TNBC) that can address the higher incidence and aggressiveness of TNBC in African-American (AA) women. Our previous studies have demonstrated annexin A2 (AnxA2) association with exosomes which promotes angiogenesis and metastasis. Therefore, our goal was to examine the expression and function of exosomal-annexin A2 (exo-AnxA2) derived from the serum samples of breast cancer patients.

**Methods:**

The expression of serum exo-AnxA2 and its association with clinicopathological features of the breast cancer patients were determined. The role of serum exo-AnxA2 to promote angiogenesis was determined by an in vivo Matrigel plug assay.

**Results:**

Our results show that the expression of serum exo-AnxA2 in breast cancer patients (*n* = 169; 83.33 ± 2.040 ng/mL, *P* < 0.0001) is high compared to non-cancer females (*n* = 68; 34.21 ± 2.238 ng/mL). High expression of exo-AnxA2 levels in breast cancer was significantly associated with tumor grade (*P* < 0.0001), poor overall survival (hazard ratio (HR) 2.802; 95% confidence intervals (CI) = 1.030–7.620; *P* = 0.0353), and poor disease-free survival (HR 7.934; 95% CI = 1.778–35.398; *P* = 0.0301). The expression of serum exo-AnxA2 levels was significantly elevated in TNBC (*n* = 68; 109.1 ± 2.905 ng/mL; *P* < 0.0001) in comparison to ER^+^ (*n* = 50; 57.35 ± 1.545 ng/mL), HER2^+^ (*n* = 59; 78.25 ± 1.146 ng/mL), and non-cancer females (*n* = 68; 34.21 ± 2.238 ng/mL). Exo-AnxA2 showed diagnostic values with a maximum AUC as 1.000 for TNBC, 0.8304 for ER^+^, and 0.9958 for HER2^+^ compared to non-cancer females. The expression of serum exo-AnxA2 was significantly elevated in AA women with TNBC (*n* = 29; 118.9 ± 4.086 ng/mL, *P* < 0.0001) in comparison to Caucasian-American TNBC (*n* = 27; 97.60 ± 3.298 ng/mL) patients. Our in vivo results suggest a role of serum exo-AnxA2 in angiogenesis and its association with aggressiveness of TNBC in AA women.

**Conclusions:**

Our results demonstrated that the expression of serum exo-AnxA2 is high in AA women with TNBC and promotes angiogenesis. These findings suggest that exo-AnxA2 holds promise as a potential prognosticator of TNBC and may lead to an effective therapeutic option.

## Introduction

In the past decade, tumor-derived exosomes (50–150 nm) have been heavily studied in cancer development, metastasis, and drug resistance. Nearly every cell type secretes exosomes, but transformed cells, on average, secrete more exosomes than healthy cells. Interestingly, tumor exosomes maintain proper compartmentalization of important micro and macro molecules that are regulators of many hallmarks of cancer [1–3]. Tumor-derived exosomes are secreted into the bloodstream and are known to manipulate the metastatic cascade through angiogenesis, signal transduction, chemo-resistance, genetic intercellular exchange, and pre-metastatic-niche formation [4–9]. Additionally, circulating tumor-derived exosomes have been identified as having potential prognostic and diagnostic significance in cancer subtypes. The standard clinical recommendation to diagnose the presence of a malignant tumor is often procurement biopsy, but this invasive standard often has detrimental effects [10, 11]. Thus, the investigation of tumor exosomes as a diagnostic or prognostic marker may offer new opportunities for a minimally invasive procedure that would adequately prognosticate and diagnose a disease progression in patients.

Triple-negative breast cancer (TNBC) lacks the three widely used diagnostic markers (human epidermal growth factor receptor 2, Her-2; progesterone receptor, PR; and estrogen receptor, ER). Thus, women diagnosed with this disease are unable to benefit from the identification of the markers for early detection, targeted therapy, and prognosis [12, 13]. Overall, TNBC is associated with poor prognosis, high mortality rate, shorter median time to relapse (due to its aggressive tumor phenotypes), high recurrence rate, and visceral metastatic spread to the brain and lungs. The disparities in breast cancer seen in African-American (AA) women may arise due to biological factors such as obesity, tissue inflammation and altered phosphoprotein signaling, and environmental causes such as unsafe neighborhoods, access to health-care treatment, low family income, stress, and exposure to environmental carcinogens [14–17]. Though life style and genetic differences are correlated with high incidence of basal breast carcinomas in AA women after adjusting for socioeconomic factors, the incidence and mortality rate remains higher than other ethnicities. This suggests that the clinical outcome of TNBC in AA women may result from biological differences. There is an urgent clinical need to identify new target(s) that can be used as diagnostic and prognostic tools and targets for therapeutic intervention that would eradicate this health disparity and provide health equity for AA TNBC patients.

Our recent studies have identified annexin A2 (AnxA2), a 36-kDa calcium-dependent phospholipid-binding protein, as one of the most highly expressed proteins in breast cancer and breast cancer exosomes [18–21]. Additionally, exosomal-AnxA2 (exo-AnxA2) expression is significantly higher in malignant cells than normal and pre-metastatic breast cancer cells [21]. Our studies in MCF10A breast cancer progression model (MCF10A, immortalized mammary epithelial cell line; MCF10AT, premalignant cell line generated by HRAS transformation of MCF10A; and MCF10CA1a, derived from poorly-differentiated malignant tumors from MCF10AT xenograft) revealed that the expression levels of exo-AnxA2 are highly associated with the aggressiveness of breast cancer cells, with lower levels in MCF10A, moderate levels in MCF10AT, and significantly higher levels in MCF10CA1a; however, the whole cell lysate analysis of the progression model revealed no significant changes in the levels of AnxA2 in MCF10AT and MCF10CA1a [21]. Interestingly, the levels of other angiogenic markers, including vascular endothelial growth factor (VEGF), urokinase-type plasminogen activator (uPA), and matrix metalloproteinase 9 (MMP9), were relatively unchanged. Our immunoelectron micrograph analysis showed that AnxA2 is predominantly present at the surface and lumen of the exosomes [21, 22]. Furthermore, our in vitro and in vivo studies demonstrated that exo-AnxA2 derived from breast cancer cells promotes angiogenesis. Additionally, our studies indicate that metastatic TNBC exosomes create a favorable microenvironment for metastasis and exo-AnxA2 plays an important role in establishing a pre-metastatic niche at the site of metastasis. This indicates that AnxA2 association with exosomes is involved in tumorigenesis and has potential to be a prognostic or diagnostic marker [21, 23, 24]. Given the fact that breast cancer cells and tumors secrete significant amounts of exosomes, we hypothesize that exo-AnxA2 from AA TNBC patients will have higher amounts of exo-AnxA2 secreted in their serum which contributes to the aggressiveness of their disease. Our efforts to establish exo-AnxA2 as an important determinant of racial disparity and disease aggressiveness in TNBC are highly innovative as this is the first study in which AnxA2 is evaluated in a race-derived patient cohort. Currently, limited information is available on potential diagnostic and prognostic markers in TNBC that can address the higher incidence and aggressiveness of TNBC in AA women in comparison to Caucasian-American (CA) women. Thus, we aimed to correlate exo-AnxA2 serum expression with AA TNBC patients and to determine the significance of elevated levels of circulating exo-AnxA2 with measures of disease aggressiveness in AA women with TNBC.

## Materials and methods

### Archived serum collection

Archived serum samples of breast cancer patients (*n* = 169) and non-cancer females (*n* = 68) were collected from the Tissue Management Shared Resource, Simmons Comprehensive Cancer Center, University of Texas Southwestern Medical Center, Dallas, Texas. The samples were stored at − 80 °C, were thawed at room temperature (RT), and immediately placed on ice prior to use. All the archived serum samples were acquired under Institutional Review Board (IRB)-approved protocols at the site of collection and the University of North Texas Health Science Center, Fort Worth, Texas. The samples were analyzed in a double-blinded study where the clinicopathological reports of the sample were not revealed to the investigator until after completion of analysis.

### Exosome isolation from serum and size analysis

Exosomes from breast cancer serum samples were isolated by using a total exosome isolation reagent (Life Technologies, USA) according to the manufacturer’s protocol. Briefly, the serum samples were thawed at RT and centrifuged at 2000×*g* for 30 min at 4 °C to remove cells and debris. This clarified serum sample (100 μL) was mixed with 20 μL of the reagent and vigorously mixed with a vortex and pipetting up and down until there was a homogenous solution. This mixture was incubated at 4 °C for 30 min. After incubation, the sample was centrifuged at 10,000×*g* for 10 min at RT. The supernatant was discarded and the exosomal pellet was resuspended in PBS for analysis. Average sizes of the exosomes were determined by a Malvern Zetasizer particle size analyzer (Malvern Instruments Ltd., Malvern, UK). The exosomal pellet was resuspended in PBS, and the size distribution was analyzed. The results were reported as the average of five runs with triplicates in each run.

### Preparation of exosome extracts and Western blot analysis

Exosomes isolated from serum samples were resuspended in radioimmunoprecipitation assay (RIPA) lysis buffer containing protease and phosphatase inhibitor cocktail (Millipore Corporation, MA) at 4 °C for 30 min. After sonication on ice, debris was removed by centrifugation at 12,000×*g* for 10 min at 4 °C. Protein concentrations were determined by BCA protein assay kit (Thermo Scientific, IL). Exosome extracts were separated on 4–20% Bis-Tris Nu-PAGE gel (Invitrogen Corporation, CA) using MES buffer and transferred onto nitrocellulose membrane. Membranes were blocked with 5% fat-free milk in Tris-buffered saline containing 0.05% Tween-20 (TBST) at room temperature for 60 min and incubated overnight at 4 °C with the appropriate primary antibody in 5% milk in TBST. After washings with TBST, the membrane was incubated with the appropriate secondary antibody (Southern Biotech, AL) at room temperature for 2 h. After washing again with TBST, the membranes were developed using ECL plus (Millipore Corporation, MA) and the image was captured using alpha-imager Fluoretech HD2. The following antibodies were used for Western blotting analysis: AnxA2 (BD Biosciences, CA), TSG101 (BD Biosciences, CA), flotillin-1 (BD Biosciences, CA), calnexin (BD Biosciences, CA), GM130 (BD Biosciences, CA), EpCAM (Cell Signaling Technology, MA), and CD9 (Cell Signaling Technology, MA).

### Exosomal AnxA2 analysis by enzyme-linked immunosorbent assay (ELISA)

AnxA2 levels in serum exosomes were analyzed by an ELISA kit (R&D systems, MN) according to the manufacturer’s protocol. Briefly, a 96-well microplate was coated with capture antibody overnight at 4 °C, washed three times, and blocked with blocking buffer for 2 h at RT. Next, the plates were incubated with serum exosomes and diluted in buffer for 2 h at RT. The plates were washed and coated with detection antibody for 2 h at RT and washed again. The plates were incubated with Streptavidin-HRP for 20 min at RT, washed, and further incubated with 3,3′,5,5′-tetramethylbenzidine (TMB) peroxidase substrate. The reaction was stopped using 2N H_2_SO_4_ and the optical density was read at 450 nm with wavelength correction at 540 nm. Samples were run in triplicate (*n* = 3).

### In vivo Matrigel plug assay

The Matrigel plug assay was performed as described previously with slight modifications [21, 25]. Briefly, 500 μL of Matrigel (BD Biosciences, CA) mixed with either PBS (negative control) or serum-derived exosomes (100 μg; pooled from 5 random patients to eliminate bias) treated with or without control peptide (LGKLSL) or AnxA2 inhibitory peptide (LCKLSL) were injected subcutaneously at the left or right lower abdominal wall of the 4- to 6-week-old athymic nude mice (Harlan Laboratories, WI). Three mice were injected for each control and experimental group. Mice were sacrificed 18–20 days after the Matrigel injections and the Matrigel plugs were recovered and photographed. Matrigels were snap frozen in liquid nitrogen for hemoglobin estimation using Drabkin’s reagent.

### Hemoglobin estimation by Drabkin’s reagent

Hemoglobin estimation from the Matrigel was performed by Drabkin’s method [26]. To quantify the formation of functional vasculature in the Matrigel plug, the amount of hemoglobin was measured using a Drabkin reagent kit 525 (Sigma, MO) following the Drabkin and Austin methods [27]. Briefly, the Matrigel plugs were homogenized in a Dounce homogenizer on ice in the presence of 0.5 mL deionized water and allowed to stand overnight at 4 °C. The lysate was centrifuged at 5000×*g* for 10 min and the supernatant was collected. Next, 0.3 mL of each sample was mixed with 0.5 mL of Drabkin’s reagent and allowed to stand for 15 min at room temperature. The absorbance was read at 540 nm by using Drabkin’s reagent solution as blank. A standard curve was constructed by using known concentrations of hemoglobin, and the concentration of the samples was obtained from the standard curve.

### Statistical analysis

GraphPad Prism 8 (GraphPad Software, CA) and SPSS software (SPSS Inc., IL) were used for all statistical analysis. Scatter plots were used to plot the serum exo-AnxA2 levels, and the results were presented as mean ± SEM. Comparison of mean between two groups was conducted using Student’s *t* test, while the comparison for more than two groups was conducted using one-way ANOVA. Data that did not satisfy parametric assumptions were analyzed using non-parametric test. Survival data of serum exo-AnxA2 were derived from clinical information for each breast cancer patient. The cutoff values for AnxA2 expression for “low” and “high” were determined using the median of the serum exo-AnxA2 concentrations in breast cancer patients. Overall survival (OS) was defined as the interval between the date of surgery and date of death from any cause. Disease-free survival (DFS) was defined as the interval from the date of surgery or treatment to the date of recurrence diagnosis. Kaplan–Meier estimation and log-rank tests were used to analyze differences in survival durations (reported using hazard ratios and corresponding 95% confidence intervals (CI)) [28]. These analyses determined the impacts of the serum exo-AnxA2 on OS and DFS. To determine whether serum exo-AnxA2 could be a potential diagnostic tool for aggressive breast cancer, receiver operating characteristic (ROC) curves were used to compare the serum exo-AnxA2 levels of cancer patients and non-cancer patients. Statistical significance was two-tailed and considered significant if *P* value was at least ≤ 0.05: (*), *P* < 0.05; (**), *P* < 0.01; (***), *P* < 0.001; (****), *P* < 0.0001.

## Results

### AnxA2 is expressed in exosomes isolated from serum samples of breast cancer patients

**Fig. 1:**
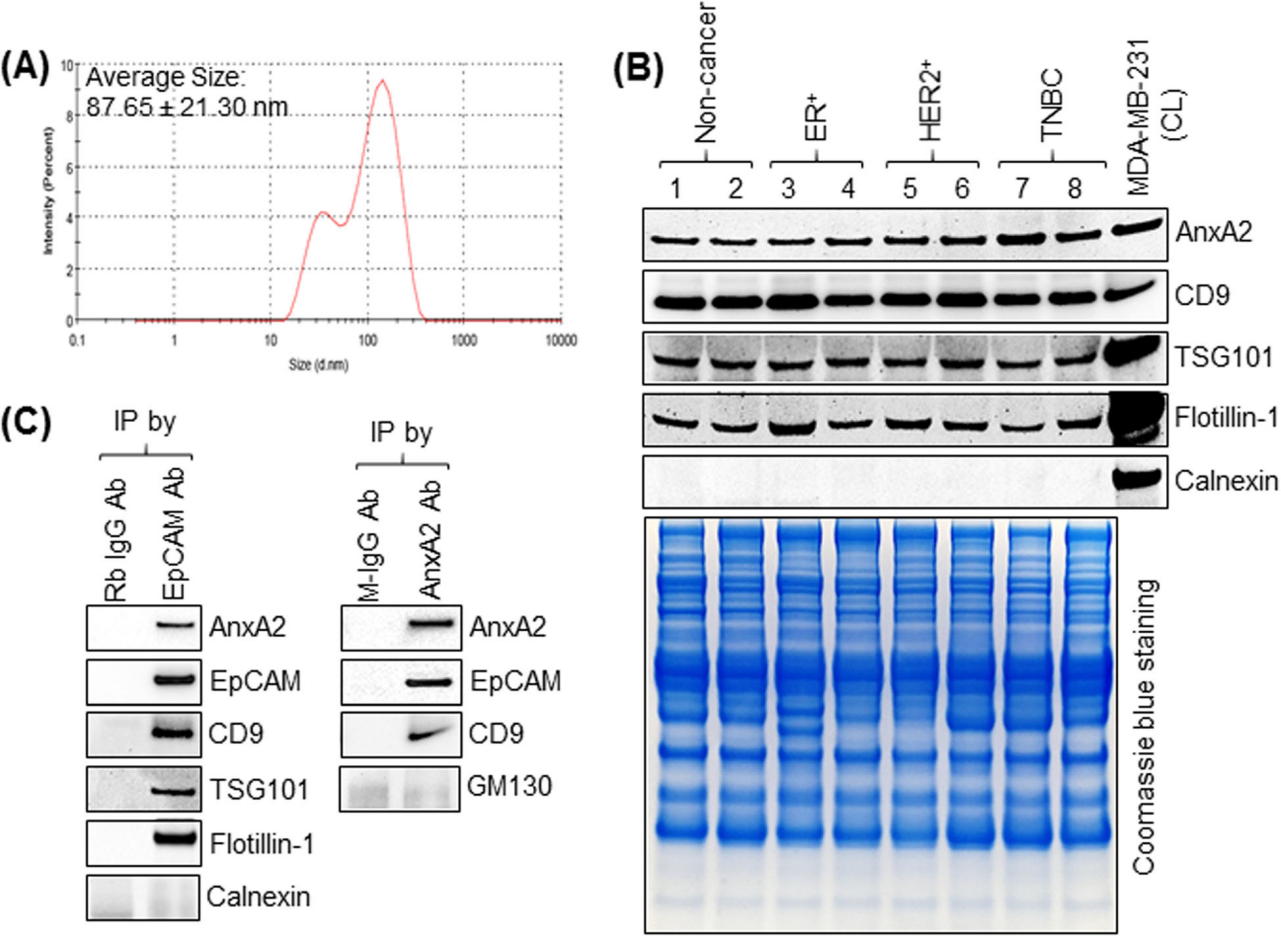
Expression of AnxA2 in exosomes derived from serum samples of breast cancer patients. **a** Size analysis of exosomes. Representative image of the average size of exosomes isolated from serum samples of cancer patients were analyzed by Malvern Zetasizer. Studied exosomes range of size is 52.06–122.3 nm in diameter with an average size of 87.85 ± 21.30 nm. **b** Western blot analysis for the expression of AnxA2 and exosomal markers CD9, TSG101, and flotillin-1 in lysates of exosomes purified from breast cancer patients and non-cancer patients. MDA-MB-231 cell lysate was used as a positive control for the expression of AnxA2, CD9, TSG101, flotillin-1, and calnexin. Calnexin (endoplasmic reticulum marker) was analyzed as a marker absent in exosomes. Coomassie blue staining was performed as an equal loading control for exosomes derived from serum samples of patients. **c** Exosomes purified from serum samples of breast cancer patients were immunoprecipitated with an antibody to AnxA2, EpCAM, or isotype mouse/rabbit immunoglobulin (M-IgG/ Rb-IgG). The immunoprecipitated whole exosomes were lysed with RIPA lysis buffer and analyzed for the expression of AnxA2, EpCAM (endothelial marker), exosomal markers (CD9, TSG101, and flotillin-1), calnexin (endoplasmic reticulum marker), and GM130 (cis-Golgi marker) by Western blot analysis. Calnexin and GM130 were used as a negative control for exosomes

We previously showed that AnxA2 is present in exosomes derived from the breast cancer cells [21]. Therefore, we investigated whether AnxA2 is expressed in exosomes isolated from the serum samples of breast cancer patients. As the first step, exosomes isolated from serum samples of breast cancer patients and non-cancer females were characterized for the expression of exosomal marker proteins and size analysis. The average size of isolated exosomes indicated that the vesicles were approximately 87.85 ± 21.30 nm in diameter (Fig. 1a). Furthermore, Western blot analysis revealed that the serum exosomes were positive for the expression of exosomal protein markers CD9, TSG101, and flotillin-1 while calnexin, an endoplasmic reticulum marker not expressed in exosomes, is negative (Fig. 1b). Our results show that AnxA2 is also present in exosomes isolated from serum samples of non-cancer females and ER^+^, HER2^+^, and TNBC breast cancer patients (Fig. 1b). To demonstrate that AnxA2 present in serum exosomes originate from epithelial cells, we performed immunoprecipitation of exosomes using anti-AnxA2 or anti-EpCAM antibodies to purify AnxA2- and EpCAM-positive exosomes, respectively. Western blot analysis show that EpCAM-positive breast cancer exosomes express AnxA2 and exosomal markers CD9, TSG101, and flotillin-1 (Fig. 1c). Similarly, AnxA2-positive exosomes also contain epithelial cell marker EpCAM and exosomal marker CD9 (Fig. 1c). In addition, EpCAM- and AnxA2-positive exosomes are negative for the expression of calnexin and GM130, respectively, and show the purity of exosomes isolated from breast cancer serum samples. These findings demonstrate that exosomes isolated from serum samples of non-cancer females and subtypes of breast cancer patients carry AnxA2 and are derived from epithelial cells.

### Serum exo-AnxA2 is associated with breast cancer

**Fig. 2:**
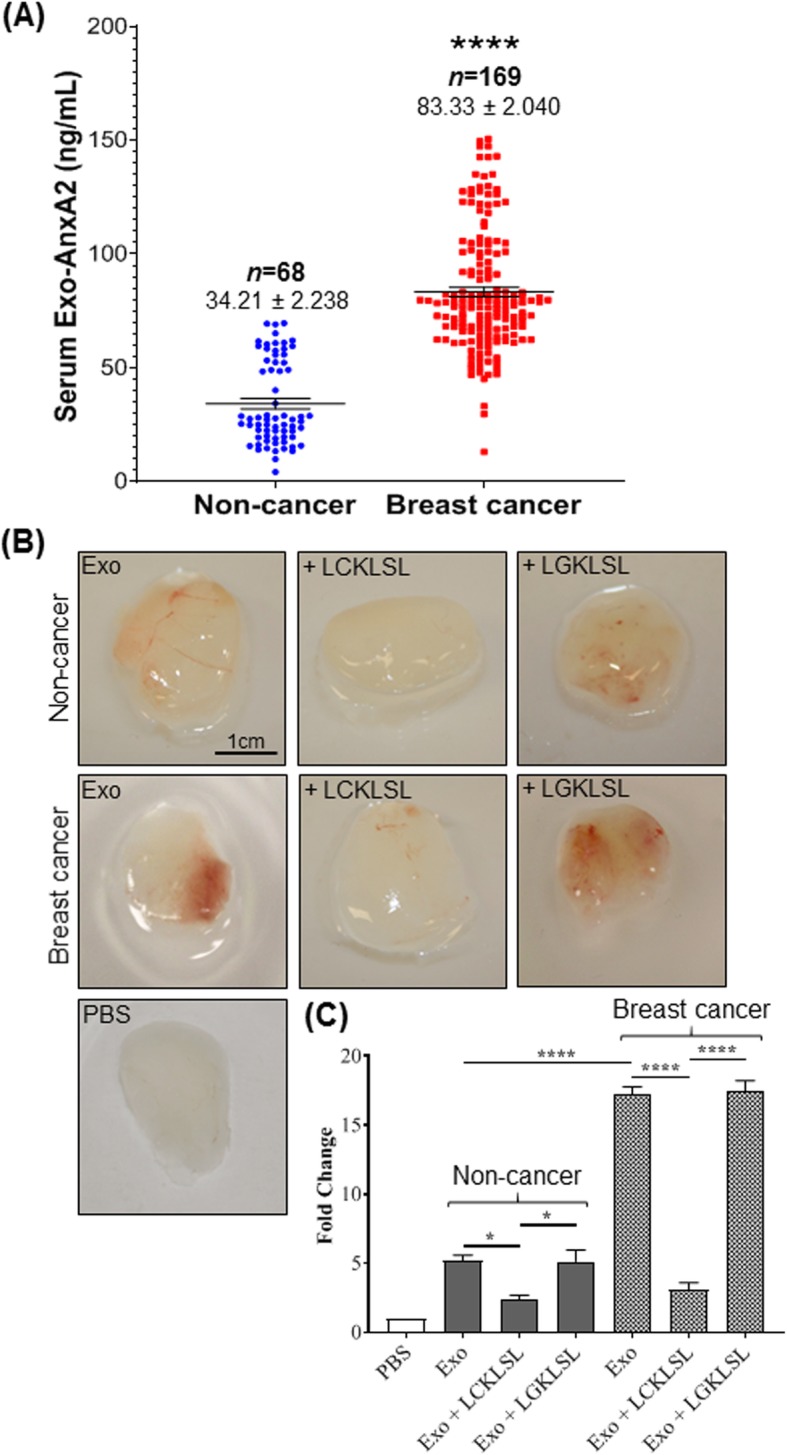
Exo-AnxA2 analysis in serum samples of breast cancer patients and non-cancer females. **a** Scatter plot analysis of exo-AnxA2 protein concentration obtained through ELISA analysis from serum of non-cancer (*n* = 68) females and breast cancer (*n* = 169) patients. Each point represents the mean of triplicates. The data are expressed as the mean ± SEM (****, *P* < 0.0001; two-tailed Student’s *t* test). **b** Matrigel plug assay with serum exosomes derived from non-cancer females and breast cancer patients along with incubation with LCKLSL AnxA2 inhibitory or LGKLSL control peptides was performed in athymic nude mice (*n* = 3). Representative images of the Matrigel plugs are shown. Peptide concentration: 5 μmol/L. **c** Quantification of hemoglobin estimation of homogenized Matrigel plugs from non-cancer and breast cancer serum exosomes by Drabkin’s method (*n* = 3; *, *P* < 0.05; ****, *P* < 0.0001; one-way ANOVA followed by Tukey’s multiple comparison test)

Having shown that AnxA2 is present in the serum samples of breast cancer patients and predominantly localized at the surface of the exosomes [19, 21, 22], we measured the circulating concentrations of exo-AnxA2 in serum samples of breast cancer patients and non-cancer females. Serum samples collected from breast cancer patients (*n* = 169) and non-cancer females (*n* = 68) were analyzed in a double-blind study for exo-AnxA2 protein levels. The demographics and health information of breast cancer patients and non-cancer females are listed in Table 1. ELISA analysis shows that exo-AnxA2 concentration was significantly elevated in serum samples of breast cancer patients (*n* = 169, 83.33 ± 2.040 ng/mL, *P* < 0.0001) in comparison to non-cancer (*n* = 68, 34.21 ± 2.238 ng/mL) females (Fig. 2a). We have previously shown that exo-AnxA2 collected from cell culture supernatants is a promoter of angiogenesis [21]. To further confirm our findings, we performed a Matrigel plug assay in athymic nude mice with exosomes collected from serum samples of breast cancer patients and non-cancer females. Gross examination of Matrigel plugs showed abundant vessel formation in the plugs containing serum exosomes and few vessels in plugs with PBS alone (Fig. 2b). However, the extent of vessel growth was significantly higher in Matrigel plugs with exosomes derived from serum samples of breast cancer patients than plugs containing serum exosomes of non-cancer females. When we incubated the serum exosomes with LCKLSL AnxA2 inhibitory peptide, we found drastic decrease in degree of vessel formation than incubation with LGKLSL control peptide (Fig. 2b); this confirms both groups of exosomes induced new vessel formation in an AnxA2-dependent manner. We further confirmed our findings by analyzing the amount of hemoglobin present in the Matrigel plugs via Drabkin’s method (Fig. 2c). The hemoglobin concentration in the plugs containing breast cancer exosomes was significantly higher (~ 3.2 fold) than those with non-cancer serum exosomes. As evident from Matrigel plug images, incubation of the plugs with breast cancer serum exosomes resulted in approximately 5.8-fold decrease in hemoglobin concentration with LCKLSL AnxA2 inhibitory peptide treatment, which did not occur with LGKLSL control peptide treatment (Fig. 2c). In addition, injection of Matrigel with non-cancer serum exosomes plus LCKLSL had similar effects (~ 2.2-fold decrease) compared to LGKLSL control peptide treatment. These findings suggest that high concentration of serum exo-AnxA2 is a potent inducer of angiogenesis in breast cancer patients.

**Table 1:** Demographics of serum samples of breast cancer patients

	ER^+^	HER2^+^	TNBC	Total
Case (%)	50 (29.94)	59 (35.33)	58 (34.73)	167 (100)
Race (*n* = 160)				
CA	25 (15.63)	38 (23.75)	27 (16.87)	91 (56.25)
AA	25 (15.63)	16 (10)	29 (18.12)	70 (43.75)
Tumor grade (*n* = 159)				
I	12 (7.55)	4 (2.51)	–	16 (10.06)
II	25 (15.72)	15 (9.44)	9 (5.66)	49 (30.82)
III	12 (7.55)	36 (22.64)	46 (28.93)	94 (59.12)

### Relationship between serum exo-AnxA2 expression levels and the clinicopathological features of the breast cancer patients

**Fig. 3:**
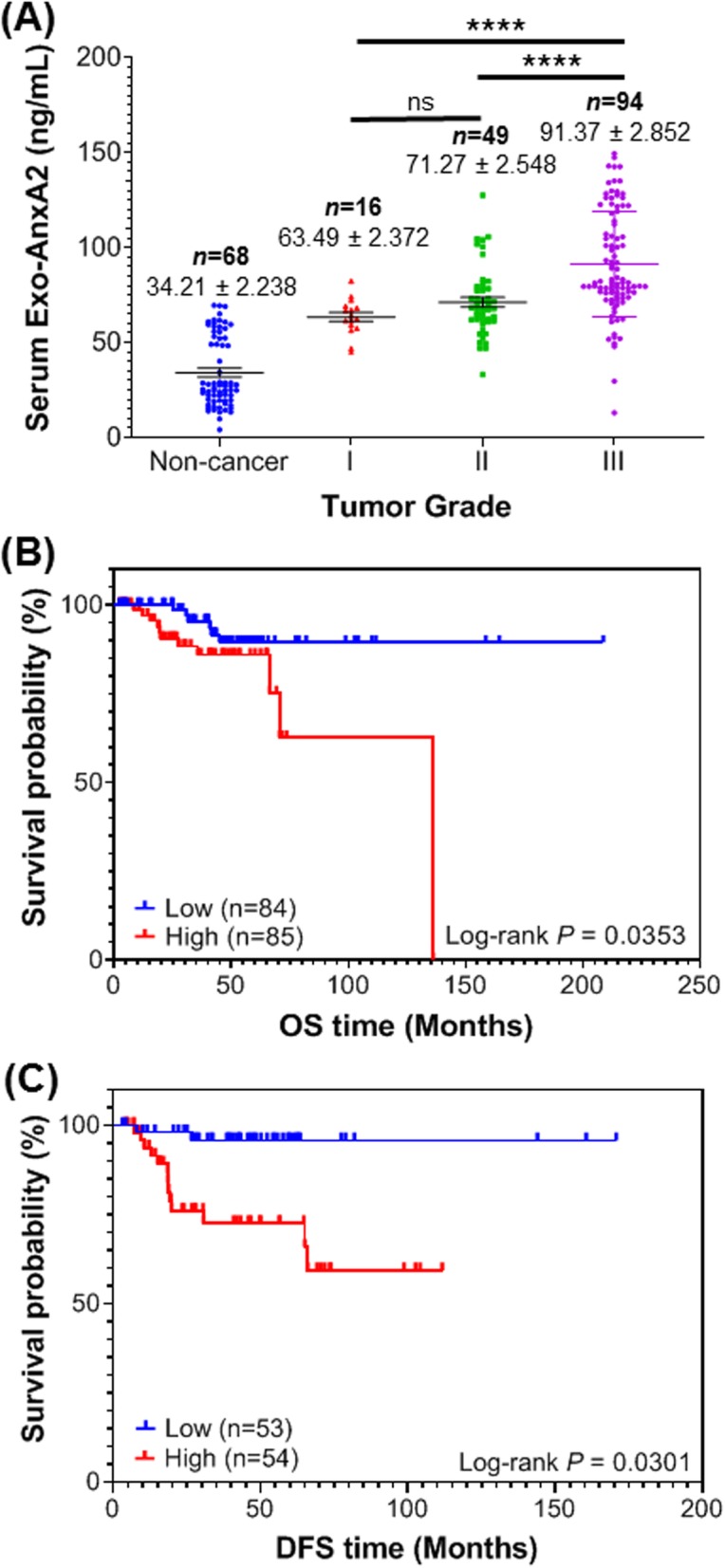
The expression of exo-AnxA2 levels and its association with clinicopathological features. **a** Scatter plot analysis of expression of serum exo-AnxA2 levels in non-cancer females (*n* = 68) and different grades of breast tumor patients (grade I, *n* = 16; grade II, *n* = 49; and grade III, *n* = 94). The data are expressed as the mean ± SEM (^ns^, non-significant; ****, *P* < 0.0001; one-way ANOVA followed by Tukey’s multiple comparison test). Kaplan-Meier survival analysis for exo-AnxA2 levels in serum samples of breast cancer patients. **b** Overall survival of patients with breast cancer based on serum exo-AnxA2 expression using Kaplan-Meier curves (*n* = 169). **c** Diseases-free survival of patients with breast cancer on serum exo-AnxA2 expression (*n* = 107). The *P* value was calculated using the log-rank test

The clinicopathological features such as tumor size, grade, lymph node metastasis, and TNM stage and its association with serum exo-AnxA2 relative expression status were examined in breast cancer patients. Significant association between breast tumor grades and serum exo-AnxA2 relative expression levels were observed (Fig. 3a) and tumor size, lymph node metastasis, and TNM stage showed no significant association with circulating levels of serum exo-AnxA2 with the progression of the disease. As shown in Fig. 3a, the mean concentration of serum exo-AnxA2 in non-cancer patients was 34.21 ± 2.238 ng/mL (*n* = 68), whereas that in patients with grade I, II, and III breast tumor was 63.49 ± 2.372 ng/mL (*n* = 16, *P* < 0.0001), 71.27 ± 2.548 ng/mL (*n* = 49, *P* < 0.0001), and 91.37 ± 2.852 ng/mL (*n* = 94, *P* < 0.0001), respectively. The concentration of serum exo-AnxA2 in grade III breast patients was significantly higher than that of grade I and II breast tumor patients (*P* < 0.0001). However, no significant difference was observed between grade I and II breast tumor patients.

### High serum exo-AnxA2 expression is correlated with poor survival in breast cancer patients

Emerging evidence shows that AnxA2 is upregulated and correlated to poor prognosis in patients with breast cancer [29, 30]. In our study, the Kaplan–Meier method was performed to analyze the relationship between serum exo-AnxA2 levels and OS of patients with breast cancer. We used the median expression value for serum exo-AnxA2 in breast cancer patients (*n* = 169) to stratify into a high exo-AnxA2 (> 77.87 ng/mL) and a low exo-AnxA2 groups (< 77.87 ng/mL). The results demonstrated that breast cancer patients with high levels of serum exo-AnxA2 (*n* = 85) had significantly shorter OS (hazard ratio 2.802; 95% CI = 1.030–7.620; log-rank *P* = 0.0353) than those with low levels of serum exo-AnxA2 (*n* = 84; Fig. 3b). In addition, we also determined the correlation between exo-AnxA2 levels in the serum and DFS of the breast cancer patients. The median expression value of serum exo-AnxA2 in breast cancer patients (*n* = 107) was used for DFS evaluation and stratified into high exo-AnxA2 (> 70.87 ng/mL) and low exo-AnxA2 groups (< 70.87 ng/mL). We found that high serum exo-AnxA2 levels (*n* = 54) were associated with worse DFS (hazard ratio 7.934; 95% CI = 1.778–35.398; log-rank *P* = 0.0301) in breast cancer patients (*n* = 53; Fig. 3c). Taken together, our survival analysis confirms that high expression of circulating exo-AnxA2 in serum results in a poor survival of the breast cancer patients and suggests that the circulating exo-AnxA2 could predict prognosis of breast cancer patients.

### High serum exo-AnxA2 is associated with African-American TNBC patients

**Fig. 4:**
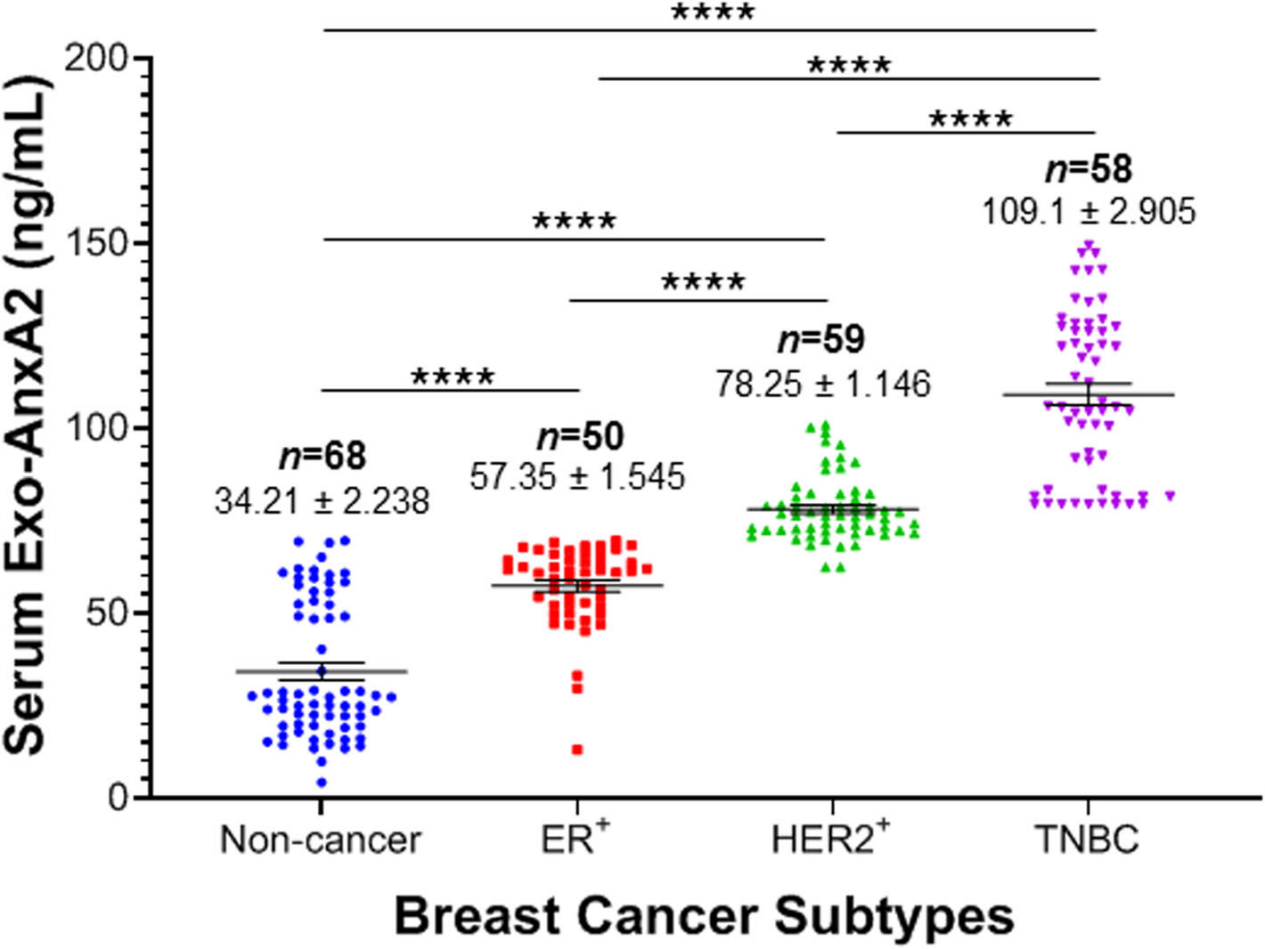
Exo-AnxA2 expression among breast cancer subtypes. Scatter plot analysis of serum exo-AnxA2 concentration in non-cancer (*n* = 68), ER^+^ (*n* = 50), HER2^+^ (*n* = 59), and TNBC (*n* = 58) breast cancer patients. The data are expressed as the mean ± SEM (****, *P* < 0.0001; one-way ANOVA followed by Tukey’s multiple comparison test)

**Fig. 5:**
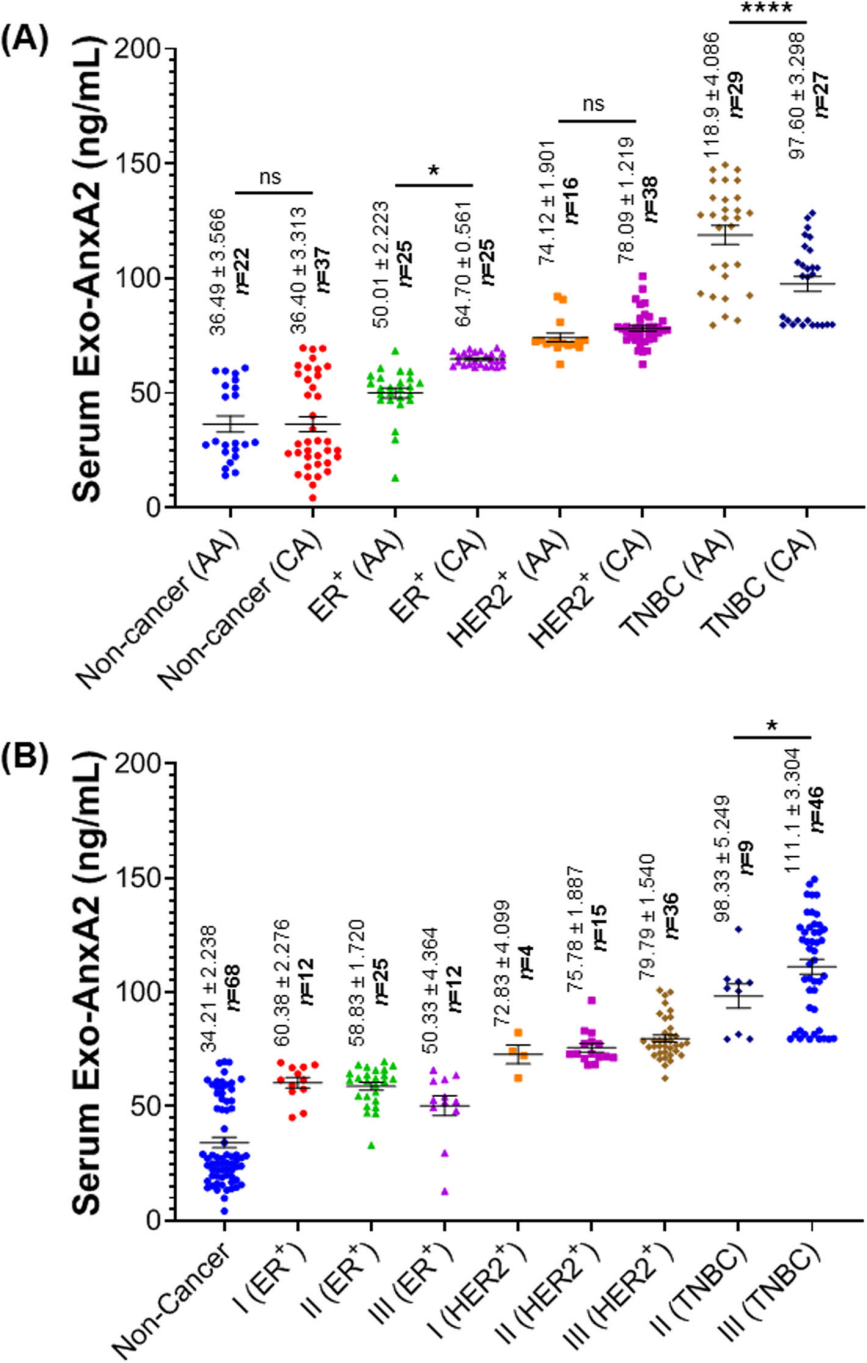
Serum exo-AnxA2 expression in breast cancer subtypes and its association with race and tumor grade: **a** The concentration of serum exo-AnxA2 expression levels among race in subtypes of breast cancer patients and non-cancer females. The data are expressed as the mean ± SEM (*, *P* < 0.05; ****, *P* < 0.0001; two-tailed Student’s *t* test). **b** Scatter plot analysis of serum exo-AnxA2 levels in ER^+^, HER2^+^, and TNBC breast cancer patients of different tumor grades. The data are expressed as the mean ± SEM (*, *P* < 0.05; one-way ANOVA followed by Bonferroni’s multiple comparison test)

In our previous studies, we have shown that AnxA2 is overexpressed in TNBC in comparison to other subtypes of breast cancer [20, 31]. Therefore, we measured the expression of exo-AnxA2 levels in serum samples of ER^+^, HER2^+^, and TNBC breast cancer patients (Table 1). Our ELISA analysis clearly suggests that the relative expression of exo-AnxA2 levels were significantly elevated in TNBC (*n* = 68, 109.1 ± 2.905 ng/mL) in comparison to ER^+^ (*n* = 50, 57.35 ± 1.545 ng/mL, *P* < 0.0001), HER2^+^ (*n* = 59, 78.25 ± 1.146 ng/mL, *P* < 0.0001), and non-cancer (*n* = 68, 34.21 ± 2.238 ng/mL, *P* < 0.0001) serum samples (Fig. 4). These observations show that the expression of exo-AnxA2 is predominantly associated with TNBC subtype. Our previous study indicates a strong association of AnxA2 expression with AA women with breast cancer and implicating AnxA2 as a contributor to the aggressive biology of TNBC [30]. Here, we further compared the expression of exo-AnxA2 levels in sera of AA and CA with breast cancer and non-cancer females. ELISA analysis revealed that AnxA2 expression is significantly elevated in serum exosomes isolated from AA TNBC (*n* = 29, 118.9 ± 4.086 ng/mL, *P* < 0.0001) patients in comparison to CA TNBC (*n* = 27, 97.60 ± 3.298 ng/mL) patients (Fig. 5a). In contrast, the concentration of serum exo-AnxA2 levels in ER^+^ patients were significantly high in CA ER^+^ (*n* = 25, 64.70 ± 0.561 ng/mL, *P* < 0.0153) compared to AA ER^+^ (*n* = 25, 50.01 ± 2.223 ng/mL) patients (Fig. 5a). However, no significant differences were observed in AA and CA patients of HER2^+^ breast cancer and non-cancer females. Data presented in Figs. 3a and 5a show that serum exo-AnxA2 levels in breast cancer patients increases with the tumor grade progression and exo-AnxA2 expression is high in serum samples of TNBC patients, respectively. Therefore, comparison analysis of relative expression of exo-AnxA2 levels in serum samples of different breast cancer subtypes with tumor grade progression were performed (Table 1 and Fig. 5b). Our analysis (one-way ANOVA followed by Dunnett’s multiple comparison test) clearly suggests that the relative expression of exo-AnxA2 levels were significantly elevated in different tumor grades of ER^+^ (grade I: *n* = 12, 60.38 ± 2.276 ng/mL, *P* < 0.0001; grade II: *n* = 25, 58.83 ± 1.720 ng/mL, *P* < 0.0001; or grade III: *n* = 12, 50.33 ± 4.364 ng/mL, *P* < 0.01), HER2^+^ (grade I: *n* = 4, 72.83 ± 4.099 ng/mL, *P* < 0.0001; grade II: *n* = 15, 75.78 ± 1.887 ng/mL, *P* < 0.0001; or grade III: *n* = 36, 79.79 ± 1.540 ng/mL, *P* < 0.0001), and TNBC (grade II: *n* = 9, 98.33 ± 5.249 ng/mL, *P* < 0.0001; or grade III: *n* = 46, 111.1 ± 3.304 ng/mL, *P* < 0.0001) patients in comparison to non-cancer (*n* = 68, 34.21 ± 2.238 ng/mL) female serum samples. In addition, our study showed that exo-AnxA2 expressions were significantly high in the serum samples of grade III TNBC patients compared with grade II TNBC patients (*P* < 0.029; Fig. 5b). In contrast, no significant difference in exo-AnxA2 levels were observed in ER^+^ or HER2^+^ breast cancer patients with the progression of tumor grades. To ensure that the relative expression of serum exo-AnxA2 is significantly higher in AA TNBC patients, the expression of serum exo-AnxA2 levels in CA and AA women were further analyzed after adjusting the tumor grades in TNBC population (Additional file 1: Figure S1). Our analysis clearly suggest that the relative expression of exo-AnxA2 is significantly high in serum samples of grade III AA TNBC (*n* = 24, 120.2 ± 4.455 ng/mL, *P* < 0.01) patients compared to grade III CA TNBC (*n* = 20, 99.16 ± 4.155 ng/mL) patients. However, no statistical difference in serum exo-AnxA2 levels were observed between grade II AA TNBC (*n* = 2; 116.6 ± 10.95 ng/mL) patients and grade II CA (*n* = 7; 93.11 ± 4.592 ng/mL) TNBC patients. Our observation of exo-AnxA2 preferential association with TNBC strongly suggests a role of serum exo-AnxA2 levels in predicting aggressiveness of TNBC in AA women.

### High expression of circulating exo-AnxA2 is associated with aggressive biology of AA women with TNBC

**Fig. 6:**
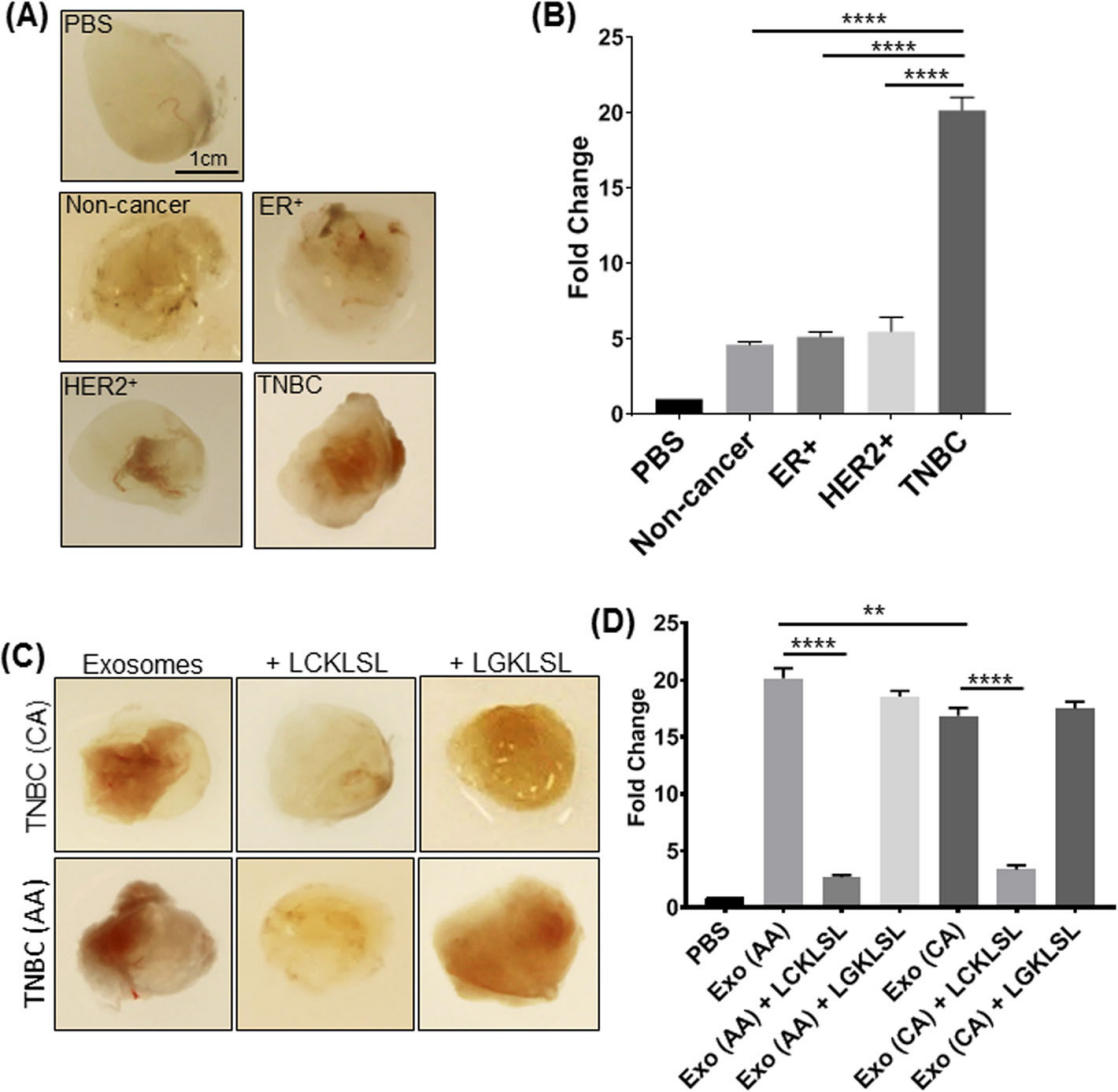
Serum exo-AnxA2 promotes angiogenesis. **a** Representative images of Matrigel plugs containing serum exosomes derived from non-cancer, ER^+^, HER2^+^, TNBC breast subtypes, and their impact on angiogenesis. **b** Quantification of angiogenesis formation through hemoglobin estimation by Drabkin’s method (*n* = 3; ****, *P* < 0.0001; one-way ANOVA followed by Tukey’s multiple comparison test). **c** Representative images of Matrigel plugs containing serum exosomes derived from AA and CA TNBC patients that show comparison of angiogenesis between AA and CA TNBC patients. LCKLSL (AnxA2 inhibitory peptide) and LGKLSL (control peptide) were used to demonstrate the functional role of AnxA2 in contributing to angiogenesis. Peptide concentration: 5 μmol/L. **d** Quantification of angiogenesis formation through hemoglobin estimation by Drabkin’s method (*n* = 3; **, *P* < 0.01; ****, *P* < 0.0001; one-way ANOVA followed by Tukey’s multiple comparison test)

The results presented in Fig. 2 suggest that the high expression of exo-AnxA2 levels is a potent inducer of angiogenesis in breast cancer patients. To further confirm that high expression of exo-AnxA2 levels in serum correlated with aggressive metastasis in TNBC, an in vivo Matrigel plug assay was performed using exosomes derived from ER^+^, HER2^+^, and TNBC breast cancer subtypes and non-cancer females. Data shown in Fig. 6a demonstrate a visible increase in vessel formation in plugs containing TNBC exosomes in comparison to other breast cancer subtypes and non-cancer exosomes. We further confirmed our observation through quantification of new blood vessel formation within these Matrigel plugs through hemoglobin estimation by Drabkin’s method [26]. Our results show that hemoglobin concentration in Matrigel plugs containing TNBC exosomes is approximately fourfold higher compared to plugs containing ER^+^, HER2^+^, or non-cancer exosomes (Fig. 6b). To further examine that aggressive metastases in AA women with TNBC is correlated with the high levels of circulating exo-AnxA2 present in TNBC patients, an in vivo Matrigel plug assay was further performed in female nude mice using exosomes derived from serum samples of AA and CA TNBC patients with or without LGKLSL or LCKLSL peptides. We visually observed attenuation of angiogenesis in Matrigel plugs containing LCKLSL AnxA2 inhibitory peptide in both CA and AA TNBC exosomes and increased angiogenesis in exosomes alone or exosomes containing LGKLSL control peptide (Fig. 6c). Hemoglobin analysis of Matrigel plugs further confirmed that CA and AA TNBC exosomes containing LCKLSL inhibitory peptide inhibits approximately 5-fold and 7.5-fold blood vessel formation, respectively (Fig. 6d), compared to their respective CA and AA serum exosomes. However, the exosomes treated with LGKLSL control peptide did not show any significant reduction in hemoglobin concentration and blood vessel formation compared to their respective exosomes alone (Fig. 6c, d). In addition, the hemoglobin content is significantly high (~ 1.2-fold) in plugs containing AA TNBC exosomes in comparison to plugs with CA TNBC exosomes. Our observation of exo-AnxA2 preferential association with AA TNBC patients suggests a potential role for exo-AnxA2 as a contributor to the aggressiveness of TNBC in AA women.

### Serum exo-AnxA2 has good diagnostic value for aggressive breast cancer

**Fig. 7:**
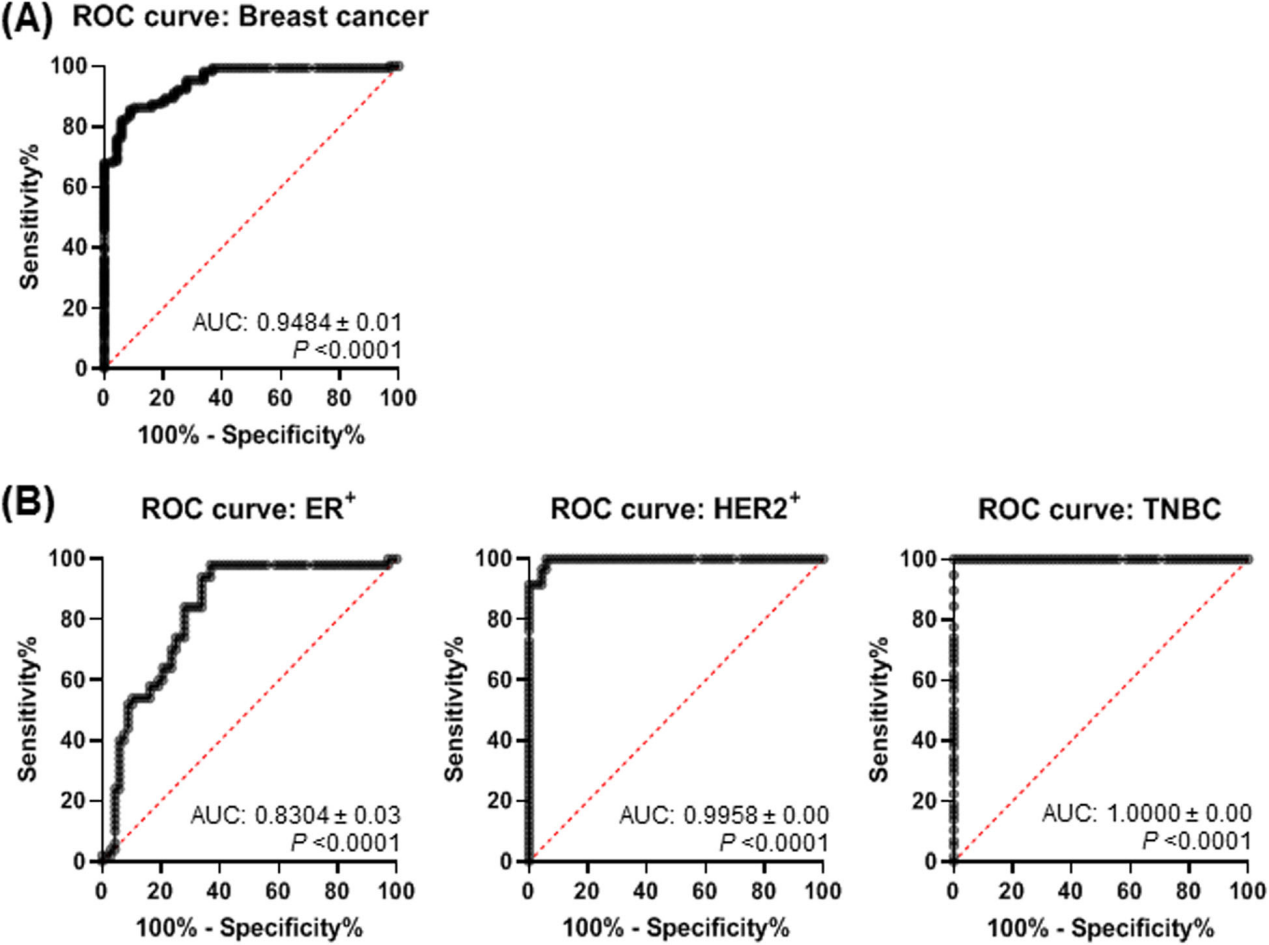
Diagnostic outcome for the prediction of aggressive breast cancer. **a** Receiver operating characteristic (ROC) curve analysis using serum exo-AnxA2 for discriminating breast cancer patients (*n* = 169) from non-cancer females (*n* = 68). **b** ROC curve analysis for discriminating ER^+^ (*n* = 50), HER2^+^ (*n* = 59), and TNBC (*n* = 58) patients from non-cancer females (*n* = 68) using serum exo-AnxA2. The AUC values are shown on the graphs

To determine whether serum exo-AnxA2 could be a potential diagnostic tool for aggressive breast cancer, receiver operating characteristic (ROC) curves were used to compare the serum exo-AnxA2 levels from 169 breast cancer patients and 68 non-cancer patients. The area under the curve (AUC) for the ROC curve of the test with exo-AnxA2 levels in serum samples of breast cancer patients as the disease indicator was 0.9484 ± 0.01327 (95% CI = 0.9223–0.9744, *P* < 0.0001; Fig. 7a). The diagnostic ability of serum exo-AnxA2 was also evaluated in ER^+^ (*n* = 50), HER2^+^ (*n* = 59), and TNBC (*n* = 58) patients compared to non-cancer (*n* = 68) patients. ROC curves of serum exo-AnxA2 in breast cancer subtypes showed that AUC values of ER^+^, HER2^+^, and TNBC were 0.8304 ± 0.03843 (95% CI 0.7551–0.9058, *P* < 0.0001), 0.9958 ± 0.0029 (95% CI 0.9899–1.000, *P* < 0.0001) and 1.000 ± 0.000 (95% CI 1.000–1.000, *P* < 0.0001), respectively (Fig. 7b). These results indicate that serum exo-AnxA2 levels might be an appropriate diagnostic tool for aggressive breast cancer specifically in TNBC patients.

## Discussion

Success in the prognosis of cancer is largely dependent on a comprehensive understanding of cancer biology and its relationship to clinical outcomes. Exosomes are precursors of metastasis that have huge potential to broaden our ability to provide adequate prognoses [32–35]. Exosome secretion of diverse biological molecules enables a variety of markers that can be analyzed to assist diagnosis and prognosis of cancer patients [6, 11, 36]. Proteomics analysis of exosomes from cultured breast cancer cells and sera of breast cancer patients identified AnxA2 as one of the vital metastatic proteins which plays an important role in angiogenesis and metastasis [21]. The expression of exo-AnxA2 was significantly higher in sera of breast cancer patients compared to non-cancer females (*P* < 0.0001). In addition, the expression of exo-AnxA2 progressively increases with tumor grades of breast cancer patients (*P* < 0.0001). The presence of AnxA2 in EpCAM-positive exosomes clearly suggests the accuracy and specificity for epithelial cancerous origins to a certain extent [37–39]. The diagnostic value of breast cancer showed that the expression of exo-AnxA2 levels in serum could distinguish the breast cancer patients (AUC value 0.9484 ± 0.01327) from the non-cancer females. High expression of exo-AnxA2 levels in breast cancer patients exhibited worse OS and DFS, suggesting that the level of serum exo-AnxA2 has an important predictive value in breast cancer prognosis.

Exo-AnxA2 has been observed in the progression and metastases of TNBC [21]. Our present study also indicates a strong association of exo-AnxA2 with TNBC in comparison to ER^+^, HER2^+^, and non-cancer females. Consistent with our present studies, we have recently found that AnxA2 protein is significantly overexpressed in tumors of TNBC patients compared to ER^+^ and/or PR^+^, HER2^+^, and normal breast tissues (unpublished data). Our previous studies have shown that phosphorylation of AnxA2 at tyrosine 23 significantly promotes the transport of AnxA2 to the plasma membrane [22]. In addition, Src kinase, which is known to phosphorylate AnxA2 at tyrosine 23, is also localized in the membrane of the TNBC [40–43]. Therefore, it is possible that the high expression of exo-AnxA2 levels seen in serum samples of TNBC patients could be due to high expression of Src kinase which is predominantly overexpressed in TNBC [43]. Here, we have also analyzed a race-specific patient cohort in a double-blind study and were able to link exo-AnxA2 to AA TNBC women. We found that exo-AnxA2 expression was higher in the sera of AA TNBC patients in comparison to CA TNBC patients (*P* < 0.0001) even after adjusting the tumor grades in AA and CA TNBC patients. The expression of exo-AnxA2 levels in serum samples of different breast cancer subtypes with tumor grades clearly suggests that the progressive increase of exo-AnxA2 levels seen in serum samples of breast cancer patients (Fig. 3a) is specifically associated with TNBC subtypes of breast cancer (Fig. 5b). Unlike TNBC, the expression of exo-AnxA2 is high in the sera of CA ER^+^ patients in comparison to AA ER^+^ patients (*P* < 0.05). However, the diagnostic value to discriminate ER^+^ patients from non-cancer females is low (ER^+^: AUC value 0.8304 ± 0.03843) in comparison to TNBC (AUC value 1.000 ± 0.000) patients. To the best of our knowledge, this is the first report on the diagnostic value of exo-AnxA2 in serum samples from breast cancer patients. The high expression of exo-AnxA2 levels in serum samples of AA TNBC patients and its association with tumor grades are consistent with our previous studies showing that the high expression of AnxA2 mRNA in tumor tissues of different breast cancer subtypes is significantly associated with the progression of TNBC and AA TNBC patients [30]. Furthermore, the racial disparity in breast cancer patients is predominantly present in TNBC and three times higher in AA women with TNBC in comparison to other ethnicities [14]. Together, this unique phenomenon may explain the association of high exo-AnxA2 with the aggressiveness of TNBC observed in AA women [14–17, 30].

The results of the present study, along with our previous study, clearly suggest that exo-AnxA2 derived from the cell culture supernatant or sera of the breast cancer patients contributes to the formation of new blood vessels [21]. The extent of new blood vessel formation is high in TNBC in comparison to other subtypes of breast cancer and more specifically in AA women with TNBC. Furthermore, we found that exo-AnxA2 is a potent inducer of angiogenesis and its effect can be blocked by a specific AnxA2 inhibitory peptide [21, 44]. These observations suggest that exo-AnxA2 facilitates neo-angiogenesis in TNBC patients and may contribute to the increase of distant metastasis seen in AA TNBC women. Thus, exo-AnxA2 not only presents itself as a potential prognostic and diagnostic marker, but also as a potential therapeutic target [21]. Further, exo-AnxA2 presents a unique opportunity for use in a minimally invasive procedure for AA TNBC patients that are often diagnosed at later stages and have higher treatment latency. In several routine blood draws over the course of a patient’s disease, we can potentially monitor cancer aggressiveness and predict clinical outcomes.

This study was significant as it detailed exo-AnxA2 association with TNBC in AA women and its contribution to the aggressiveness of the TNBC disease. Despite the relevance and innovation of this study, there were several limitations. First, TNBC only makes up 10–15% of all breast cancer cases and is often difficult to acquire serum samples in large numbers, especially from AA women. Further, our low number of patients in TNBC did allow for any significant correlation of exo-AnxA2 levels to clinical outcomes such as age, TNM stage, metastatic sites, menopausal status, relapse, and mortality [45–48]. Our full understanding of serum-derived exo-AnxA2 and its association with metastasis would be a seminal discovery that would allow the opportunity to the clinician to provide the appropriate therapeutic option. Additionally, we would like to understand the exo-AnxA2 relationship with other ethnicities and ancestry to better understand its association with the disproportionate occurrences in incidence, mortality, metastasis, and relapse seen within these patients. In conclusion, exo-AnxA2 holds promise as a potential prognostic predictor that can be analyzed in a non-invasive procedure in AA TNBC patients and may lead to an effective therapeutic option.

## Conclusion

Triple-negative breast cancer (TNBC) affects women of African descent three times more than women of European descent [14]. It is critical to investigate the molecular mechanism(s) that lead to aggressive disease in AA women with TNBC so that improved therapeutic options can be developed. Here, we show that the expression of exo-AnxA2 is elevated in the sera of the breast cancer patients and plays an important role in angiogenesis. The high expression of exo-AnxA2 was associated with tumor grade, poor overall survival, and disease-free survival of breast cancer patients. The serum exo-AnxA2 level was upregulated in TNBC patients with high diagnostic value in comparison to other subtypes. In addition, the association of exo-AnxA2 expression with tumor grade of breast cancer patients is specifically associated with the triple-negative subtypes of breast cancer. The aggressiveness of TNBC in AA women is linked with the high expression of exo-AnxA2 levels present in their serum. The detection of serum exo-AnxA2 levels could be useful for the diagnosis, prognosis, and therapy for AA women with TNBC.

## Supplementary information

Additional file 1: **Figure S1.** Serum exo-AnxA2 expression in CA and AA women after adjusting the tumor grade in TNBC population. Scatter plot analysis of serum exo-AnxA2 levels in CA and AA TNBC patients of grade II and III tumors. The data are expressed as the mean ± SEM (*, *P* < 0.01; one-way ANOVA followed by Tukey’s multiple comparison test).

## Data Availability

All remaining data and materials are available from the authors upon reasonable request.

## Abbreviations

AA: African-American
AnxA2: Annexin A2
AUC: Area under the ROC curve
CA: Caucasian-American
CI: Confidence interval
DFS: Diseases-free survival
ELISA: Enzyme-linked immunosorbent assay
ER: Estrogen receptor
Exo-AnxA2: Exosomal-annexin A2
HER2: Human epidermal growth factor receptor type 2
HR: Hazard ratio
OS: Overall survival
PR: Progesterone receptor
ROC: Receiver operating characteristic
TNBC: Triple-negative breast cancer
