# Correction to: Serum exosomal-annexin A2 is associated with African-American triple-negative breast cancer and promotes angiogenesis

**DOI:** 10.1186/s13058-020-01268-9

**Published:** 2020-03-23

**Authors:** Pankaj Chaudhary, Lee D. Gibbs, Sayantan Maji, Cheryl M. Lewis, Sumihiro Suzuki, Jamboor K. Vishwanatha

**Affiliations:** 1 Department of Microbiology, Immunology and Genetics, Graduate School of Biomedical Sciences, University of North Texas Health Science Center, 3500 Camp Bowie Blvd., Fort Worth, TX 76107 USA; 2 Simmons Comprehensive Cancer Center, University of Texas Southwestern Medical Center, Dallas, TX 75390 USA; 3 Department of Biostatistics and Epidemiology, School of Public Health, University of North Texas Health Science Center, Fort Worth, TX 76107 USA; 4 Texas Center for Health Disparities, University of North Texas Health Science Center, Fort Worth, TX 76107 USA

**Correction to: Breast Cancer Research (2020) 22:11**

**https://doi.org/10.1186/s13058-020-1251-8**

After publication of the original article [1], we were notified that the wrong version of Fig. 2b has been published.

Below the correct version of Fig. 2b”.


