# Supplementary figures and images for: Serum exosomal-annexin A2 is associated with African-American triple-negative breast cancer and promotes angiogenesis
Source: Breast Cancer Res. 2020 Jan 28;22:11. doi: 10.1186/s13058-020-1251-8 (PMC6986157; doi:10.1186/s13058-020-1251-8)

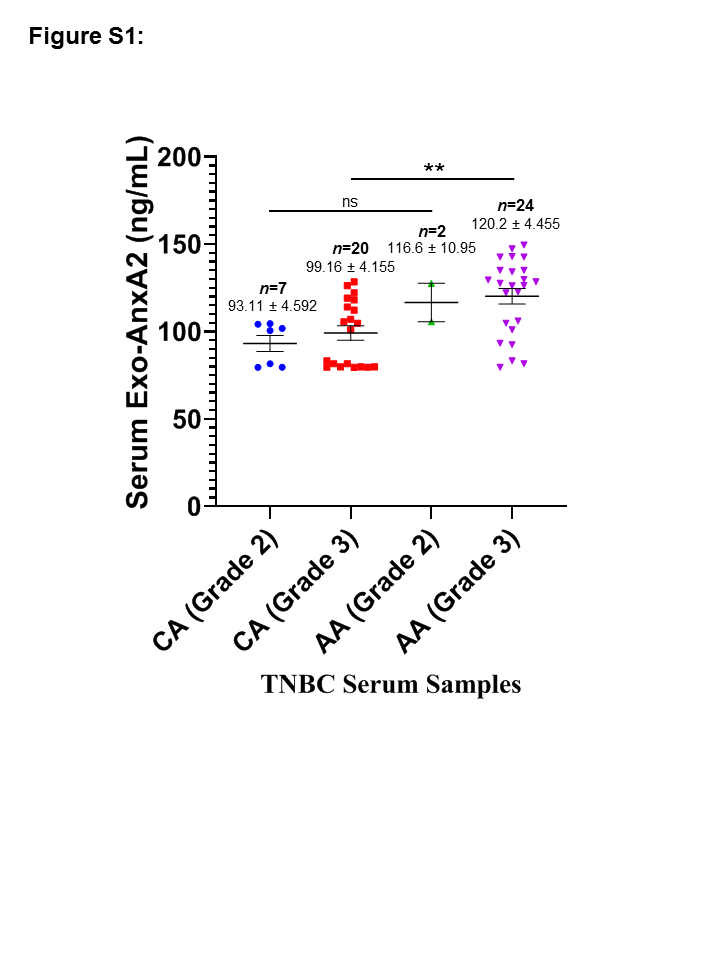

Supplement: Supplementary file 1 — Figure S1. Serum exo-AnxA2 expression in CA and AA women after adjusting the tumor grade in TNBC population. Scatter plot analysis of serum exo-AnxA2 levels in CA and AA TNBC patients of grade II and III tumors. The data are expressed as the mean ± SEM (*, P < 0.01; one-way ANOVA followed by Tukey’s multiple comparison test). [file 13058_2020_1251_MOESM1_ESM.tif]
